# Complete Genome Sequence of *Sphingopyxis macrogoltabida* Type Strain NBRC 15033, Originally Isolated as a Polyethylene Glycol Degrader

**DOI:** 10.1128/genomeA.01401-15

**Published:** 2015-12-10

**Authors:** Yoshiyuki Ohtsubo, Yuji Nagata, Mitsuru Numata, Kieko Tsuchikane, Akira Hosoyama, Atsushi Yamazoe, Masataka Tsuda, Nobuyuki Fujita, Fusako Kawai

**Affiliations:** a Department of Environmental Life Sciences, Graduate School of Life Sciences, Tohoku University, Sendai, Japan; b Biological Resource Center, National Institute of Technology and Evaluation, Tokyo, Japan; c Center for Fiber and Textile Science, Kyoto Institute of Technology, Kyoto, Japan

## Abstract

*Sphingopyxis macrogoltabida* strain 203, the type strain of the species, grew on polyethylene glycol (PEG) and has been deposited to the stock culture at the Biological Resource Center, National Institute of Technology and Evaluation (NITE), under the number NBRC 15033. Here, we report the complete genome sequence of strain NBRC 15033. Unfortunately, genes for PEG degradation were missing.

## GENOME ANNOUNCEMENT

*Sphingopyxis macrogoltabida* strain 203 was isolated from soil as the polyethylene glycol (PEG)-utilizing *Flavobacterium* sp. strain 203 (1). Later, the strain was designated the type strain of *Sphingopyxis macrogoltafidus* (2) and deposited to the Institute for Fermentation, Osaka (IFO) (Osaka, Japan) under the number IFO 15033, which was reidentified as *S. macrogoltabida* (3) based on the taxonomical standards proposed by Yabuuchi et al. (4). Since IFO was closed, the strain was transferred to the National Institute of Technology and Evaluation (NITE) and has been stocked under the number NBRC 15033.

The genome of NBRC 15033 was sequenced using 454 GS-FLX Titanium (Roche) and HiSeq systems (Illumina). A fragment library was constructed for 454 GS-FLX sequencing, and we obtained 98,820 reads (69 Mb). For Illumina HiSeq sequencing, mate-pair and paired-end libraries were constructed and sequenced for 151 bp from both ends. ShortReadManager (SRM) was used to extract long-distance mate-pair (MP) reads from the mate-pair data. SRM was also used to trim the MP reads based on the occurrence frequency data of 21-mers, which were obtained from the paired-end data of approximately 1,000-fold coverage. Thus, rarely occurring parts of reads that derived from the sequencing error or sequence adapter were trimmed. We used Newbler version 2.8 to assemble the 454 reads and trimmed mate-pair reads. The total number of bases used for assembly was 390 Mb, and we obtained 179 contigs and three scaffolds. The finishing was facilitated by the computer programs GenoFinisher (http://www.ige.tohoku.ac.jp/joho/gf_e/) and AceFileViewer (5), and all gaps were closed by *in silico* analyses. The finished sequence was confirmed by FinishChecker, which is an accessory tool of GenoFinisher.

The sequence was annotated by the NCBI Prokaryotic Genomes Annotation Pipeline (PGAP), and the resulting annotation was subjected to manual correction by using the annotation support tool of GenomeMatcher (6). In the correction, MiGap annotation (http://www.migap.org/) (7) was used to correct the start codon positions and to add genes missing in the PGAP annotation.

The complete genome sequence comprised one circular chromosome with a size of 5,174,928 bp and two plasmids with sizes of 422,455 bp and 151,240 bp. The *peg* operon for the degradation of PEG was located on a megaplasmid (8). However, the *pegA* gene encoding PEG dehydrogenase (9, 10) was missing in the finished genome sequence, and only a part of the *peg* operon (accession number AB239080) was present on the chromosome. Flanking transposons and an insertion sequence located next to *pegA* (8) might have been involved in the loss. The strain had been repeatedly cultured on nutrient broth since it was deposited to IFO, and the strain might have lost the PEG-degrading ability. We recovered a strain harboring the *pegA* gene that was capable of growing on PEG from a laboratory stock. This strain has been deposited to the culture collection at NITE as another strain named 203N, and its genome sequencing is now under way.

### Nucleotide sequence accession numbers.

The genome sequence of *S. macrogoltabida* strain 203 has been deposited in the NCBI under the accession numbers CP009429 to CP009431.
